# System interventions to support rural access to maternity care: an analysis of the rural surgical obstetrical networks program

**DOI:** 10.1186/s12884-023-05898-7

**Published:** 2023-08-29

**Authors:** Jude Kornelsen, Stephanie Lin, Kim Williams, Tom Skinner, Sean Ebert

**Affiliations:** 1 Centre for Rural Health Research, Department of Family Practice, University of British Columbia, 5950 University Boulevard, 3rd Floor David Strangway Building, Vancouver, British Columbia V6T1Z3 Canada; 2 Faculty of Land and Food Systems, University of British Columbia, British Columbia, Vancouver, Canada; 3 Rural Coordination Centre of British Columbia, Vancouver, Canada

**Keywords:** Rural maternity care, Health service planning, Health services accessibility, Rural health, Qualitative research

## Abstract

**Background:**

The Rural Surgical Obstetrical Networks (RSON) project was developed in response to the persistent attrition of rural maternity services across Canada over the past two decades. While other research has demonstrated the adverse health and psychosocial consequences of losing local maternity services, this paper explores the impact of a program designed to increase the sustainability of rural services themselves, through the funding of four “pillars”: increased scope and volume, clinical coaching, continuous quality improvement (CQI) and remote presence technology.

**Methods:**

We conducted in-depth, qualitative research interviews with rural health care providers and administrators in eight rural communities across British Columbia to understand the impact of the RSON program on maternity services. Researchers used thematic analysis to generate common themes across the dataset and interpret findings.

**Findings:**

Participants articulated six themes regarding the sustainability of maternity care as actualized through the RSON project: safety and quality through quality improvement opportunities, improved access to care through increased surgical volume and OR backup, optimized team function through innovative models of care, improved infrastructure, local innovation surrounding workforce shortages, and locally tailored funding models.

**Conclusion:**

Rural maternity sites benefited from the funding offered through the RSON pillars, as demonstrated by larger volumes of local deliveries, nearly unanimous positive accounts of the interventions by health care providers, and evidence of staffing stability during the study time frame. As such, the interventions provided through the Rural Surgical Obstetrical Networks project as well as study findings on the common themes of sustainable maternity care should be considered when planning core rural health services funding schemes.

**Supplementary Information:**

The online version contains supplementary material available at 10.1186/s12884-023-05898-7.

## Background

There has been extensive research in the past two decades on the attrition of rural maternity services in Canada [1–3] and internationally [4, 5] alongside documentation of the adverse health and social consequences of losing local services [6–8]. Less attention, however, has been paid to evidence to support interventions to stabilize these services, and less still to evaluations of such interventions. The Rural Surgical Obstetrical Networks initiative in British Columbia (BC), was an innovative response to the attrition of rural maternity services based on recognition of the need for local access to caesarean section. This involved a coherent approach to sustaining rural low-volume surgical services, because caesarean sections alone would not provide the procedural volume required to ensure that operating teams maintained skills and professional confidence. Sustainable rural health care requires a commitment to generalism, meaning that rural health care providers are trained to provide the full breadth of clinical and emergency services for the community as well as enhanced surgical skills training to perform a suite of low-acuity procedures on low-risk patients. Maintaining rural generalist providers, including physicians, nurses and midwives, is necessary to sustain core services in small hospitals and to prevent the cascading effect of losing surgical, maternity and emergency services which can lead to high provider turnover and recruitment challenges.

Work evaluating the only comprehensive Enhanced Skills training program for Family Physicians in Canada found that although training was available to support an expansive scope, most enhanced surgical skills (ESS) physicians limit their care to core procedures (appendectomy, herniorrhaphy, caesarean section and colonoscopy) [9] and that outcomes, particularly of caesarean sections by ESS demonstrate safety when performed by Family Physicians with Enhanced Surgical Skills (FPESS) compared to obstetricians (OBs) [10–14]. This does not, however, address the challenge of sustainability in a low-volume setting [15] which this paper sets out to do from the perspective of providers and administrators in rural communities. In this way, it contributes essential evidence on how to sustain rural maternity services through a systematic and coherent approach.

The Rural Surgical Obstetrical Networks (RSON) initiative in British Columbia was funded by the Joint Standing Committee on Rural Issues, a provincial joint clinical committee representing collaboration between the provincial Ministry of Health and the professional association, Doctors of British Columbia. Funding was made available between November 2018 and April 2023 to support evidence-based system interventions to sustain rural, small volume maternity and procedural care, in the case of maternity care, namely caesarean section. Local access to caesarean section increases the proportion of the population that can safely birth in the community and mitigates stress for local providers [10, 16]. The funding was applied initially to eight communities representing different geographies and health authorities; three additional communities joined the program after the initial start date. The total cost of the RSON project, across all RSON communities and over the course of the 5-year project, was $19.3 million dollars. RSON communities ranged in population size from 3,700 to 18,000, with the corresponding number of deliveries ranging from 54.8 to 249 annually.

The four funding streams, or “pillars” of the RSON initiative include increased scope and volume, clinical coaching, continuous quality improvement (CQI) and remote presence technology. Increased scope and volume funding was implemented to assist local hospital teams by supporting additional nursing lines and other staff required to increase the volume of local surgical programming. A key attribute of the RSON funding was the flexibility afforded to local sites to use funding in the most advantageous way for their site, while retaining the overall intent of the funding.

RSON’s approach is informed by recognition of the key relationships between the availability of surgical services and sustainable maternity care [17]. Although quality care can be provided without immediate access to caesarean section [16], the burden of isolation and lack of immediate support for potential complications compounds the stressors of providing care and can lead to burn-out and, in some instances, attrition of health care providers [18]. Pearson et al. (2020) interviewed family physicians practicing in two types of rural communities: those that offer obstetrical care and those that do not [19]. All 8 physicians in the study cited surgical backup as a key factor in sustaining obstetrics.

## Methods

This study used open-ended qualitative research interviewing and focus groups to understand the antecedents to sustainable maternity care in RSON communities, from the perspective of local care providers (nurses, physicians and midwives) and administrators over four years of the RSON program. The study was conducted in accordance with the guidelines and regulations of the University of British Columbia’s Behavioural Research Ethics Board (Ethics ID: H18-01940).

### Setting and participants

This study included 169 participants practicing in eight communities across rural BC offering procedural care, including low acuity surgery and caesarean sections, as part of the Rural Surgical Obstetrical Networks initiative. Participating communities were selected by each regional health authority based on the relative need to stabilize services and the feasibility of implementing RSON supports (increased scope and volume funding, clinical coaching, continuous quality improvement and remote presence technology). The Local Community Coordinator at each site assisted the study team with the recruitment of Family Physicians with Enhanced or Obstetrical Surgical Skills (FP-ESS/OSS) and Family Practice Anesthetists, nurses, midwives, health services administrators, local care coordinators, operating room managers, booking clerks, and medical device reprocessing technicians.

### Data collection

Data collection was longitudinal over the course of the initiative and took place between February 2019 and May 2022 either in person at hospital boardrooms or virtually on Zoom’s videoconferencing platform. All interviews were led by the principal investigator (JK) and attended by a research assistant for fieldnote taking. Prior to the interview, oral or written informed consent was obtained from all participants, including permission for audio recording. Interviews ranged in length from 17 min to 1 h 18 min (45 min on average). Both interviews and focus groups followed an interview guide that was tailored to each community’s unique successes and challenges. Interviews were transcribed by an external transcriptionist. All transcripts underwent a thorough quality assurance process to ensure accuracy, and participants who opted to view their transcript received them through email.

### Data analysis

A method of thematic analysis was used to analyze the transcripts and inductively identify themes from the complete dataset [20]. Researchers immersed themselves in the data through multiple readings of the interview transcripts and listening to the audio recordings. An open coding process then began, with researchers breaking down ideas and discrete concepts from the text into “codes” [20]. The PI and project coordinator independently developed initial codebooks for all of the data recorded to ensure consistency in data interpretation, and organized their codes into hierarchical order. Upon comparison of the two codebooks, there was a high degree of similarity, allowing them to be merged [21]. The combined codebook was then used as the basis for comprehensive coding of transcripts from across all sites, although additional codes were added in subsequent years to capture the nuances of the changing contexts. Researchers met consistently through this process to continue reviewing and revising the coding framework and improve coding reliability.

Through familiarity with the dataset, researchers identified sustainable maternity care as a meaningful thematic topic. Within this category, researchers generated sub-themes by iteratively grouping and comparing the relationships between codes, and searching for broad, overarching ideas within the dataset related to maternity care [20]. Themes were derived through engagement with the data rather than through the application of an external theoretical framework. Sustainable maternity care, presented here, was one of the meta-themes generated through researcher engagement with the data. Other meta-themes, explored in separate publications, include ‘local team function’, ‘regional relationships’, and ‘the role of generalist anesthesia’, all of which increased understanding of the effect of the RSON intervention on the stability of local services.

### Methodological rigour

Researchers were central to the active process of theme generation, and as such, it was important that researcher positionality be discussed. Reflexivity was considered, including researcher academic and social positionality, and cultural background, in order to monitor and minimize possible bias [21]. To further improve the credibility of the analysis, authors used researcher triangulation in developing the codebook, with a blend of independent and joint development. Authors also engaged the quality strategy of “persistent observation” of the data, returning to the transcripts through the analytic process in order to ensure any interpretations were representative of participants’ experiences.

## Findings

A total of 169 participants were interviewed over the course of this study, through 143 individual interviews and 9 focus group interviews. A proportionate breakdown of all study participants by professional designation is provided in Table 1.

**Table 1:** Proportional breakdown of participant professional designation

Professional Designation	Number of Participants
Family Physicians	39
Family Physicians with Enhanced Surgical or Obstetrical Skills	14
Family Practice Anesthetists	10
Hospital Administrators	30
Nurse Educators	4
Registered Nurses	56
Registered Midwives	12
Other	4
	**Total**: 169

### Emergent theoretical framework description

**Fig. 1:**
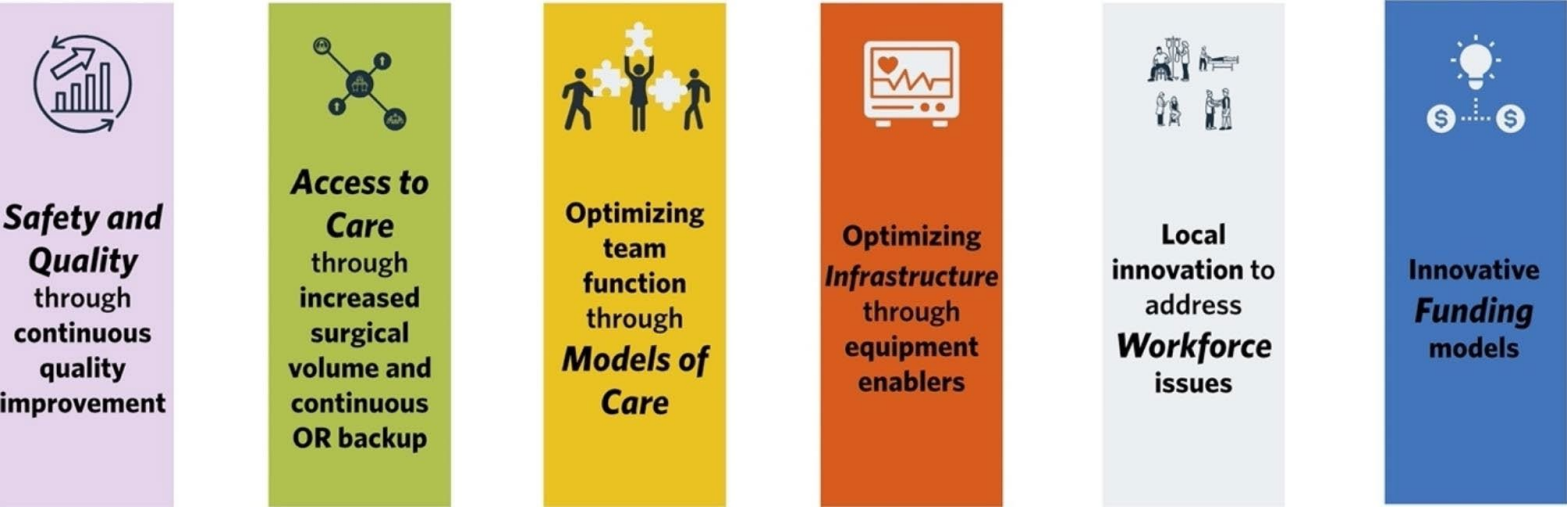
Correlates of Sustainable Rural Maternity Care actualized through the Rural Surgical and Obstetrical Networks Initiative

Participants in this study observed that the RSON initiative pillars (increased scope and volume, clinical coaching, continuous quality improvement (CQI) and remote presence technology) worked to create a comprehensive approach to stabilizing local access to maternity care. This gave rise to synergy between the original RSON pillars which coalesced in six discrete priority areas needed to sustain care. These priority areas are represented in Fig. 1, below.

Key interventions noted by participants in this study to stabilize maternity services in their communities aligned with the individual pillars of the RSON initiative and include *safety and quality through Continuous Quality Improvement*, *access to care through increased surgical volume and continuous OR backup, optimizing team function through models of care, optimizing infrastructure through equipment enablers, local innovation to address workforce issues and innovative funding models*. Each is described in detail, below.

### Principle 1: safety and quality through continuous quality improvement

An array of continuous quality improvement (CQI) initiatives designed to improve the efficiency and sustainability of maternity care were successfully implemented across most RSON sites and ranged from provider-focused initiatives (such as improving team function) to patient-focused initiatives (case reviews, developing of educational material). These initiatives aligned well with many of the communities who had been working within the Managing Obstetrical Risk Efficiently (MORE^OB^) quality program.

One CQI initiative that spread to multiple sites focused on reducing time of “decision to incision” for emergency caesarean sections. Providers who participated in the projects identified unnecessary delays in their current emergency caesarean section processes during interdisciplinary meetings and simulations and prioritized increasing efficiency to improve patient safety. CQI initiatives involved participating in simulation drills, developing patient flow mapping to maximize consistency and optimizing team cohesion by clarifying roles and responsibilities, with the ultimate objective of streamlining the transition of labouring patients to the OR, in cases when an emergency caesarian section is required.

Other CQI initiatives were focused more on intrinsic team functioning, such as improving cross-over communication during nursing shift changes through the development of site-specific protocols such as “huddle” groups and the use of whiteboards to convey essential patient information. When asked about the benefits of these initiatives, one participant indicated that when they “walk in the room to relieve someone, they could have a quick glance at [the whiteboard] and know exactly where the mom is at, what is happening, and what concerns there may be. All without having to necessarily interrupt the team” [Registered Nurse 001]. Many participants agreed that these efficient pathways of communication between maternity providers have helped to optimize workflow in the maternity ward and has contributed to improving overall team function.

Case reviews were also prioritized at several sites, focusing on debriefs of challenging cases that had occurred but also discussion of upcoming potentially ‘high risk’ cases, to ensured strategic preparedness. Case reviews often focused on instances of breech deliveries, pre-term labour, and complex neonatal stabilization. Most participants expressed that case reviews were essential to quality care. For those patients who did need to leave the community for more advanced care, some sites implemented projects aimed at reducing transport times and gathering evidence about the actual costs of transport to aid in decision making. Such projects informed participants’ decision making about patient transfer by ensuring social as well as clinical evidence informed decisions. Social evidence includes not only the transportation costs incurred when traveling for care outside the community, but also the psychosocial implications of leaving family and friends, and at times, the anxiety that this precipitates. Case reviews involving referral specialists improved triage and ensured patients received the right care from the right provider at the right time in the right place.

To improve the postpartum care in their communities, several CQI projects were implemented to focus on improving patient education, particularly for breastfeeding and caesarean section aftercare. Some participants felt that the current information provided to patients upon discharge failed to cover all of the common challenges new parents can face when returning to their homes. To improve the quality of patient education, several participants took part in CQI projects that involved the development of take-home brochures for maternity patients. One participant noted they felt

*like we don’t inform our moms very well on what to do when they go home. We give them a lot of verbal instructions when they are extremely fatigued and have hormones running amok through their bodies, so I didn’t expect they would remember. So, we created a survey, we implemented it. I called c-section patients at home to ask how they felt about how well prepared they were to go home, and then we realized there was a gap and we made this brochure.* [Registered Nurse 002]

Overall, participants in this study felt that the scope of maternity-centered CQI projects resulted in increased provider confidence in their ability to provide safe, efficient maternity care in rural communities. Many participants agreed that the increased volume and exposure to difficult maternity procedures increased their knowledge and skillsets as generalist providers. One participant shared that

*the knowledge of the maternity nurses has gone up 500 times, and it is really much better than before when we were all a bit sloppy with the way we did some of those things, and now I feel like we’re just overall functioning as a much more professional group.* [FP-OSS 001].

CQI projects also played a role in improving team function, as many participants stated that implementing new protocols and participating in drills as a team has allowed for structured conversations to help clarify the roles of all the maternity providers and ensure “everybody is all on board” to complete the task at hand.

Maternity simulations have helped care teams across the RSON sites improve their team function through shared dynamic learning experiences that increases the team’s confidence and competence with challenging maternity cases. One participant noted: “I’ve got a better relationship with my nurses… than I had last year, and maybe it’s all those sims I was doing” [FP-ESS 002]. Improvements to team function would not have been possible without the attendance of entire maternity teams, as running a drill as a full staff unit gave participants the opportunity to ensure everyone “was on the same page” and “practice as if it were real life”. Several participants stated that the high turnouts for maternity simulations can be attributed to RSON funding that provided remuneration for all attendees. Many participants said that receiving payment for training activities has

*made a huge difference in enjoyment and attendance, like people actually come so then we learn and we get to practice and we feel good. And being compensated to do those during your working hours reduces resentment and fatigue.* [Family Physician 003].

### Principle 2: Access to care through increased surgical volume and continuous OR backup

In the context of this study, access referred to ensuring the availability of on-site maternity care, primarily through continuous local access to caesarean section. Enablers of increased access are described below.

A sustainable rural maternity service must be fully equipped to provide birthers with continuous access to perinatal services in their home communities. Due to the expansive geography in British Columbia and attendant inclement weather for a large part of the year, birthers encountered challenges to travelling during labour. The consequences were significant: as one participant noted, “if they missed their ferry, they would have had to stay home to have the baby [without any health care support]” [Midwife 001]. Participants also shared concerns for patients driving during severe weather conditions, as one participant admitted they were “worried sick about their patients who drive in from [outlying community]” and “come screaming down the highway in labor and go to the hotel across the road” [Midwife 001]. Another participant shared that they

*just had friends leave the community to deliver their babies, and one left the community by private vehicle and she was ten centimetres [dilated] in her car when she got to [referral community]. So for me this was just getting too personal and affecting people in such a grave way that I was just fed up with it.* [Registered Nurse 003].

While the OR closures had a substantial impact on local access to care, many participants reported that the RSON project helped their sites to improve sustainability, especially through the stabilization of their surgical services. Many participants identified the interconnectedness of maternity and surgical services and explained that birthers and providers alike will often avoid delivering in a community without caesarean section backup. When asked about RSON’s impact on stabilizing surgical services for caesarean section, one provider said that they

*couldn’t think of anything else that singly has had the same impact on improving the quality of care that RSON has for them. Obstetrics is what keeps our operating room going. Yes, we do orthopedic surgery, but that OR is there to provide a service for the women in labour. We would not be delivering babies here if we didn’t have the operating room. So, to make sure that we are safe, we’re efficient, we have skilled nurses, staff, that’s what you need.* [Family Physician 004].

Several participants identified improved emergency OR coverage as one of the key developments across RSON sites that allowed surgical services to expand. Prior to RSON, participants explained that many providers had onerous call schedules that were leading to burnout, especially for those who were shouldering the burden of call alone. With RSON funding, many sites were able to increase their staff as well as provide compensation for call, which allowed sites to “fill big gaps in coverage” and “avoid diversions or closures due to a lack of nursing staff”. When asked about the surgical capabilities at their site, one participant explained that the increased capacity for caesarean section was one of RSON’s greatest achievements.

*It has led to 24/7 OR coverage, which was one of the goals from the very start. We got rid of this crazy bypass system […]. You know, combining that with onboarding a midwife, it’s just been such a great success.* [Family Physician 005].

### Principle 3: optimizing team function through models of care

In rural communities that support operative delivery, models of care rely on a unique combination of providers that may include midwives, family physicians, labour and delivery or generalist nurses, family practitioners with anesthesia training (FPAs), and family physicians with enhanced surgical skills (FPESSs) to ensure a greater proportion of population needs for birthing services are met [11]. This health care provider mix supports complimentary skillsets to meet community needs. Almost all participants in this study noted that their teams of interdisciplinary providers were effective at sharing on-call responsibilities, providing continuity of care for patients, and providing backup during challenging deliveries.

Many participants noted that midwives supported their physician colleagues by easing the burden of their hospital call schedules to allow for a more sustainable work-life balance, as well as provided home visits for those who gave birth locally. Participants explained that having an expanded maternity care team has led to fewer gaps in coverage and has allowed for improved continuity of post-partum care, as physicians and midwives gained comfort with seeing each other’s patients when necessary. Some participants noted that while there was some hesitancy from midwifery clients towards having a physician oversee their post-partum care instead of a midwife, many reported that “[midwives and physicians] are not all that different”. This may have been in part due to the positive inter-professional relationships developed between the providers, characterized by open pathways of communication and a sense of mutual respect. When asked about their relationship with the local physicians, one midwife shared that their

*experience has been blissful- I have become friends with almost all of the doctors and we go rafting together and we hang out. So when I go into work, I am friends with the nurses and the doctors and it is lovely. I sometimes just go into work to see people. And I know that I can ask them questions without feeling [incompetent]. I think they, for the most part, respect my expertise. Some more than others. But… I feel valued, I feel supported.* [Midwife 002].

While skepticism from some physicians towards their midwifery colleagues remained, almost all physician participants saw the value in the services that midwives provide to their communities, and some made an effort to step outside of their comfort zones and provide backup for home births. Many physician participants gained a new perspective on midwifery practice after participating in collaborative care and felt that gaining hands-on experience alongside a midwife “made it very easy for people to appreciate their skill sets and get a better understanding of midwifery. And we are very fortunate in the model we have here. It has not worked out in too many other places as well as I think it works here” [Family Physician 004]. Many physicians reported that midwives have been “a great asset [to the team]” and “fit right in without a single complaint”.

While midwives have brought great value to rural models of maternity care, rural hospitals in BC are also dependent on Family Physicians with Enhanced Surgical Skills (FPESS), Family Physicians with Obstetrical Surgical Skills and Family Practice Anesthesia (FPA) providers. FPESS providers provide critical low-acuity obstetrical and gynaecological procedures in RSON communities beyond caesarean sections, including intrauterine device insertions and hysterectomies, while FPAs provide the epidurals and general anesthesia required to makes these procedures possible. While specialists are not easily sustained in RSON communities due to their narrow scopes of practice and low case volumes characteristic of rural practice, participants noted that FPESS and FPA providers who are willing to work within a smaller scope of practice and live in the community are vital to sustaining local maternity care. One participant explained that

*we need to make space for family physicians with surgical skills, who will live here. Who can provide caesarean sections here. None of these 10 surgeons in [referral community] are going to drive here to do a caesarean section.* [Family Physician 006].

Many participants stated that the FPESS model of care has eliminated the need for the majority of patients to travel to seek maternity care from specialists in regional referral centers, an essential marker of better care for the population.

Due to the low case volumes in RSON communities, many participants partook in frequent training and travelled to referral or urban centers in order to maintain their obstetrical skillsets. Despite the extra effort required to maintain their confidence and competence, participants acknowledged their key role in upholding sustainable rural maternity services. As one noted,

*the hospital and the group as a whole are motivated to do [obstetrical care] and they understand the trickle down complications of losing an OB program, you know, if you lose your anesthetists and you lose your backup coverage in ER*. [FPA 001].

To make this collaborative care possible, many midwifery and physician care providers in this study agreed to take significant cuts to their volume and remuneration in order to support this mixed model of care. Several participants had difficulties navigating the division of call responsibilities and birth volumes amongst their colleagues due to the personal financial consequences of having fewer deliveries in a small rural population. One participant noted that since the “fees went up significantly for people providing obstetrical services and because [the midwife] took such a big chunk of our obstetrical work, for those two years I paid to do the obstetrics in [the RSON] community” [Family Physician 007]. Despite the financial consequences of sharing an obstetrical practice, participants were still willing to give up a portion of their practice because they recognized the importance of maintaining a stable obstetrical program and ensuring choice in place of birth.

### Principle 4: optimizing infrastructure equipment enablers

Many participants reflected that the physical infrastructure across RSON communities, including equipment and the hospital space, requires upgrades to support a robust maternity service backed by reliable surgical services. When RSON was first implemented in 2018, many participants identified upgrades to their facilities as a key priority for RSON funding. As one participant noted, “[it is] one of the biggest issues in [my community]” [FPA 002]. Others voiced frustrations with the lack of prioritization for upgrades to their small rural hospitals. One participant explained:

*We tend to get people offering us their used equipment as if we’re the poor cousins. And that doesn’t sit really well with me or probably most people. If it’s something we need and it’s for the benefit of the patients in the community that we serve, we shouldn’t be saying ‘well we can do it but we’re going to use a piece of equipment that’s 20 years old because [referral community] got new stuff’. And now we’re on trend to make do because their old stuff is still newer than our old stuff.* [Administrator 001].

Many participants identified their recovery rooms as the area in need of the most improvement due to their limited capacity, distance from delivery rooms, and their lack of adequate equipment. They felt that their recovery rooms were “so disconnected from everything else” and noted that this presented barriers to efficient care when nurses were forced to move between wards (and often floors) to treat labouring and post-partum patients simultaneously. One participant stated:

*I just think [having the recovery room] a little bit closer would be nice. Especially to the critical care departments. Because I always worry that when our recovery room becomes separate, is that recovery room nurse going to be alone? Or [are they] going to be on call with the second? With this new expansion when you’re so far away, you could yell forever…* [Registered Nurse 004].

Participants were also concerned with the lack of appropriate equipment available in the recovery room, including beds, intubation kits, and other equipment necessary for emergency preparedness. Some participants saw the lack of sufficient equipment as a consequence of the COVID-19 pandemic, which has required unanticipated re-allocation of resources to prioritize COVID-19 patients. Some participants were concerned about the loss of beds and equipment to accommodate COVID-19 patients, with some participants seeing the down-stream effects on maternity patients.

Despite the equipment shortages and delayed upgrades for the hospital infrastructure, participants noted that RSON provided essential funding to recruit clinical coaches. Participants who were able to receive this type of mentorship agreed that it was very beneficial to their workflow and has helped them to “point out the little habits” that were preventing them from using the space efficiently. Many participants felt that these coaching opportunities have provided them with the organizational tools they need to “adjust to maximize the small amount of area they have” to ultimately provide better maternity care. At the start of the RSON project, clinical coaching was offered primarily through specialist providers. As the project iteratively adjusted and adapted to local needs though, clinical coaching grew to fund peer coaching by local family physicians who had gained additional skills, and also nursing and midwifery coaching.

While several RSON communities are still challenged by the limited physical space at their hospital sites, many participants indicated that RSON supported the purchase of new equipment such as fetal monitoring equipment and resources to optimize the care of premature newborns. While these additions have been helpful at improving the capacity for maternity care across RSON sites, many participants agreed that the most valuable equipment purchase thus far has been the iPads that support the use of real time virtual supports (RTVS) and inter-hospital communications. Some participants were able to use iPads to speak with COVID intubation patients from outside of negative pressure rooms without the need for extensive personal protective equipment, saving nurses valuable time that could be put towards patient care. iPads have also allowed care providers to access BC’s virtual supports such as Child Health Advice in ReaL-time Electronically (CHARLiE), which connects rural physicians with on-call pediatricians who provide critical neonatal advice, and the Maternity and Babies Advice Line (MaBAL), which connects providers with rural midwives and FPs with peers with maternity expertise who provide consultations. These lines are part of a larger provincial real-time virtual support initiative funded by the Ministry of Health and the Joint Standing Committee on Rural Issues. Many participants saw immense value in MaBAL and CHARLiE, as they have allowed solo providers to access specialist or peer advice in real time “if they are concerned with a baby, so they can show the pediatrician exactly what they are looking at, which has been super helpful". One participant felt that the ability to connect with a CHARLiE pediatrician, who could see their patient on the screen and guide them through difficult cases, was “life changing” [Family Physician 006].

RSON also funded Remote Presence Technology (RPT), mobile telehealth equipment with far-end camera control that allows providers to receive real time advice from colleagues remotely. While many participants felt that the iPads were useful tools that worked well to facilitate consultations between rural providers and specialists, some found RPT’s high resolution cameras and its ability to connect with other diagnostic tools to be superior, as “it makes a difference [to see] much more of the patient, not just their face on the fuzzy iPad” [Registered Nurse 005]. Some participants have used RPT to connect with their obstetrics colleagues at the regional referral centers when CHARLiE pediatricians were not available. Many providers were also appreciative of the opportunity to learn from their specialist colleagues through RPT consultations. As one participant said:

*Going from a time where we didn’t have educational support to now being able to collaborate and provide education from pulling in people who can speak to what we know is our shortcoming and teach us about these areas is amazing, it’s fantastic. The connectivity it’s created, being able to talk to specialists through virtual technology and access people for coaching, it’s just like completely upped our game and provided so much support.* [Registered Nurse 002].

RPT also enabled education to rural maternity providers across RSON sites through its use in simulation drills. Many participants partook in interdisciplinary simulations focused on caesarean section, post-partum hemorrhage, and other complicated birth cases and felt that it was “a really valuable tool for learning” to increase their emergency preparedness. When asked about the details of their recent maternity simulations, one participant noted:

*We try to run them in as much real time as possible for most of them. Some of them are walk throughs and with others you have somebody acting like the patient- you’re trying to do things in real time and do things for real. We open up packages, we sacrifice materials for the sake of learning and afterwards have a debrief with pizza. It’s really good because a lot of the time it’s those situations that everyone lives in fear of, you know? Like a pregnant lady and an MVA coming in or stuff like that.* [Registered Nurse 006].

Despite many participants’ satisfaction with RPT and its use for consultations and simulations, RPT remains underutilized at several RSON sites. Some sites have low levels of engagement with RPT due in part to the learning curve (“just another thing that I had figure out how to do”) and its addition to their busy clinical schedules. Other participants shared that their lack of participation in RPT consultations or drills is simply because they had not had a chance to engage with the technology as of yet, and remained open to doing so once an opportunity arises.

### Principle 5: local innovation to address workforce issues

The recruitment and retention of maternity care providers, including nurses, FPAs, FPESSes, FPs, and midwives, is a current challenge across rural - and urban - Canada. RSON sites in this study were no exception. Several participants mentioned that FPAs have been particularly difficult to recruit and retain due to the “anaesthesia crisis across the country”, which has caused “the competition to attract FPAs to get a little stiff”. Participants explained that one of the largest barriers to recruiting FPAs is the lack of volume new providers would receive in rural hospital sites, as FPs “cannot survive on the ESS or the anesthesia work alone- you still have to do the rest of the family practice work too to make a living”. This makes full time FPA practices at larger regional centers more appealing to providers who wish to practice at a broader scope and volume. The loss of FPA and FPESS providers to larger centers is a threat to small community hospitals, as one participant explained that when “you lose your ESS for lack of volume and you lose your anesthesia for lack of opportunity, then now your bed’s made right?” [FPA 001], and the risk of diversions or closures of rural maternity services increases.

Many participants indicated that disparities in pay between FPAs and other specialties have also presented a challenge for recruitment across the province. One participant shared that

*it’s been hard to attract Canadian grads because the fee schedule is not competitive with other ones and that discrepancy exists with surgical and cardiology, family medicine, like everybody where it’s actually driving a crisis of this magnitude- I mean we’re short something like 25, 30 percent anesthesiologists, just for the current workload…* [FPA 003].

Most participants noted the remuneration disparity between Family Practice obstetrics and other providers with added competencies, one noting, “obstetrics providers are not paid at the same rate that hospitalists and other FPs with added competencies are” [Family Physician 008]. At times, this has created friction between disciplines.

While pay disparities persist between rural FPAs and practitioners from other disciplines, participants have come up with other strategies that have helped increase the recruitment and retention of FPAs and FPESSs, including targeting “home grown” physicians; those who already work in the community and are familiar with the unique context of rural medicine. Several participants had success with convincing their FP colleagues to pursue extended training in anesthesia or surgery to support maternity services, and happily provided the mentorship required for these physicians to gain exposure to the field. These partnerships have led to long-lasting coaching relationships that worked to create reliable networks of FPAs and FPESSs for advice or backup. One participant agreed that “recruitment best happens through education. A lot of people I know are where they are because they’ve trained there or they met people who they trained with” [Family Physician 009]. Others hoped that by offering training for local and out of province FPs they could create strong working relationships that would entice the trainees to put down roots in their communities.

Another strategy participants used to improve retention of FPAs and FPESSs is to set up coaching pairings, in which a retiring FPA or FPESS provides mentorship to a newly trained physician until they can manage their role as a solo provider comfortably. Some participants explained that the retiring physician would close their clinical practice while remaining on call for anesthesia or surgery while the new physician began to absorb their clinical and surgical volumes over time. This allowed the retiring physician to “phase out” over a period of several months to several years, while giving the incoming provider the surgical backup required to increase their confidence when working independently. While this may result in a temporary surplus of maternity providers, one participant explained that

*even if we overload the [maternity] service for a period of time where some of those physicians might not want to work so much in the last couple of years, then these younger people can kind of take it over and we won’t let the service fall down.* [FP-OSS 010].

Participants agreed that this type of health human resource redundancy amongst maternity providers has supported successful succession planning for rural physicians and improved the retention of new FPA and FPESS providers in their communities.

An additional issue that continues to impact the sustainability of health human resources is the nation-wide nursing crisis, which has resulted in nursing shortages across all of the RSON sites. Participants noted that maternity nurses are vital to the rural maternity services, as “no matter how skilled the physician or midwife is, they can’t do maternity care without the nursing support” [Family Physician 011]. A lack of nursing staff has threatened accessible maternity care at several RSON sites, which were forced to go on diversion because they could not fill their nursing lines. Participants explained that they are still experiencing “huge gaps in labour and delivery experienced nurses”, and the nurses they do have are “getting pulled in all directions because the other areas are also short staffed. So they don’t have time to do the business of maintaining our ward and skills” [Family Physician 012]. While several RSON sites did not have high enough birth volumes to warrant a full maternity nursing line, many participants stressed the need of having two designated maternity nurses per shift who would have consistency in their role and not share responsibilities with other departments. Participants agreed that having designated maternity nurses would increase provider confidence in assisting with deliveries and allow for simultaneous and effective labour and postpartum care.

Some participants thought that the most effective strategy to increase nursing resources was to first focus on retaining their current maternity nurses before hiring new staff. These participants had seen an outflux of maternity nurses to public home care positions, which were less stressful roles that allowed for a greater work life balance. In order to retain nurses in hospital lines, participants stressed that the maternity nursing positions must become “more inviting” by re-assessing scheduling and line structures to avoid overburdening shifts and call schedules. Nursing positions were also made more enticing by offering coaching and skill maintenance opportunities through frequent maternity simulations.

Ultimately, participants were committed to have their nursing lines filled with local candidates to provide stable maternity care, as opposed to transient agency nurses that provide gap coverage. Some participants shared that their sites have “more agency nurses than actual local nurses”, who stay in the community for a limited time and often don’t have the chance to form meaningful relationships and forge trust with the other maternity providers. While some participants believed that agency nurses have provided a strong short-term solution to their nursing shortage, filling nursing lines and gaining administrative support to add more full-time equivalents (FTE) positions is essential to meeting the community need for maternity care.

### Principle 6: innovative funding models

Participants expressed the importance of funding for system interventions to support maternity care, applied flexibly at a local level in response to need. Beyond enabling a well-functioning, high-quality service, dedicated funding was also seen as a show of respect to the providers working in rural communities. This is discussed further, below.

The protected funding for scope and volume, clinical coaching, remote presence technology, and continuous quality improvement provided through the RSON project was “of undisputed value” to many participants and has supported major achievements in stabilizing maternity care across all the RSON sites. Most participants attributed the increased sustainability of their maternity services to RSON and agreed that it “has been the reason why we can continue to provide the services that our community needs” [FP-ESS 013]. The dedicated scope and volume funding supported the hiring of new staff, funded visiting specialists to perform surgeries at RSON sites, and funded call groups that were previously sustained through the dedication of a few providers. One participant highlighted that “other specialists would not be coming here without the [RSON] funding. […] It’s helping the [RSON community] keep me too” [Family Physician 014]. The protected clinical coaching funding was also of importance to participants, who explained that the funding helped reimburse coaches for the time they spent on call or performing consults and ultimately allowed providers to be properly compensated for their work. One participant explained that

*having the funding for coaching and networking, I think it’s enabled some relationships to solidify and has given people incentive which is so so important. And it’s really important to be able to compensate people for the work that they’re doing, especially when they are so busy and they have so many other jobs...* [Midwife 003].

Participants also made good use of the RPT funding stream, which enabled small rural hospitals to receive the equipment necessary to support maternity simulations, telehealth, and virtual consultations. Maternity simulations were also supported by the continuous quality improvement funding, which many participants saw as the most valuable protected funding made available through RSON. Care providers agreed that being able to participate in collaborative maternity drills, case studies, and various other projects allowed their teams to gain confidence, competence, and build a healthy team dynamic.

An important characteristic of the RSON funding was its flexibility for participants to “work with [the funding] in a way that makes sense for our community” [Midwife 003]. Many participants agreed that the RSON funding was empowering and gave rural practitioners autonomy over the decisions made at their sites, as the funding was not “prescribed, like every site must do this, which is usually what comes down the pipeline” [FPA 004]. This allowed participants to form decision-making focus groups with their peers to collaboratively decide on how to utilize the funding for each of the pillars in a way that would make the greatest impact on the services at their specific sites.

While most participants were happy with the flexibility of the RSON funding, there were a few participants who, early on, found it difficult to determine which of the funding pools their project would be eligible for and felt that RSON did not provide enough structure or guidance for project implementation. Participants were very appreciative of the role that their Local Community Coordinator played in helping to identify the correct funding opportunities to support innovative maternity projects. Some participants reported conflict amongst their fellow providers when deciding on which projects to prioritize and were frustrated that maternity projects were not supported as much as projects for other disciplines were. One participant pointed out that

*a lot of the focus is on the OR surgical sustainability, which is certainly super important, but then some of the maternity doctors get their nose out of joint because they feel that there’s not enough focus being placed on the maternity nurse aspect and they feel that that’s equally important.* [FPA 005].

Perhaps the most significant indicator of the importance of flexible, community-driven infrastructure funding is the concern participants expressed regarding the end of the project. As one participant noted, it is “a scary concept for our sites” [Family Physician 015]. Concern was expressed regarding the lack of attendance at coaching drills or CQI project discussions without the monetary compensation provided by RSON, while others saw higher-level challenges: “[Without] funding, we lack the ability to affect change at a higher level, or to be listened to”, as “we haven’t felt like the health authority has been engaged or really investing in keeping some of this going” [Family Physician 016].

## Discussion

The evidence-based RSON interventions foreground key system planning imperatives that stabilize low-volume rural maternity services to achieve the mandate of providing maternity care ‘close to home’ for the majority of the population. The scaffolding around such services is the availability of adequate, dedicated and protected funding that can be directed at a local level to support the most vital program initiatives in each community, underscored by the recognition of variation between communities. The accumulation of centralized solutions struggles with “voltage drop” once actualized far from where decisions and plans are made. That is, one size does *not* fit all. Despite this, there are few other examples of funding streams allowing this level of flexibility and variability due in large part to centralized health care decision-making and the need for consistency to underscore measures of accountability [22]. The provincial funding provided for the RSON program significantly bridged the gap between the rhetoric of care ‘closer to home’ and the need to support rural care providers to provide such care [23]. Without this scaffolding, unsupported providers are more likely to step back from providing maternity care than make uncompensated time commitments to quality improvement or education and have difficultly securing coaching and mentorship due to the lack of compensatory resources for those doing the coaching. Furthermore, the direct support for high functioning teams allowed them to organize around tasks, strategize and find solutions to problems and design opportunities for teams to improve function in highly critical acute care – such as in the OR, emergency room, delivery room and critical care. The resources applied to support this project were both significant when contrasted to the historical lack of funding for rural health services and non-remarkable in the context of the $23.9 billion dollar provincial budget for health care [24]. Further work on the cost-outcomes of funding local services is forthcoming from this team and will provide further understanding of cost-benefit proposition at the heart of well-supported rural health care. Regardless, funding opens the door to greater access for rural populations which, above an economic calculation, is an equity and access commitment enshrined in the Canada Health Act and, perhaps more importantly, the social responsibility of our health care systems.

Under-resourcing of rural obstetric services has been noted in the literature [3]. Although initially, funding for physical infrastructure and technology was not included in the RSON funding model, in some cases, funding was flexible enough to accommodate these needs. However, as funding was not specifically dedicated to capital costs and infrastructure, budgetary availability was based on surplus from other funding lines, and not guaranteed. Going forward, this funding line needs to be prioritized as it underscores the capacity of local sites to not only expand practice but also to maintain a desirable service level. Within these funding constraints, the additional infrastructure that was made available through RSON, such as increased health human resources, Remote Presence Technology and other equipment upgrades pushed many of the rural sites closer towards acceptable resourcing. These resources had a ripple effect of then supporting local services through, for example, well-support quality improvement initiatives with the dedicated infrastructure to move findings directly into site-level practice. Additionally, stabilizing the rural maternity care workforce through clinical coaching and other opportunities for continuing professional development - let alone the increased staffing lines - dynamically created the conditions for further success. Ultimately, the biggest gain reported was funding for a local community coordinator to organize funding and opportunities to meet community needs.

Reflective of existing literature, successful rural models of care need to be supported and recognized as more than ‘smaller urban’ units [25]. Characteristics supported by the RSON program included not simply tolerating variability between communities in response to changing historical, contextual and health human resource influences, but recognizing this variability as an asset to be protected. That is, the capacity to adapt to changing circumstances and local population level need is a crucial health system response and a distinct rural advantage due to relative smaller size and relationship-based teamwork characteristic of rural sites. Integrated, highly functioning teams with generalist skillsets can adapt and respond in real time to patient needs. When quality activities are embedded in team function, there is an infrastructure for skill development and clear communication that supports optimization of the care team.

Accounts of the interventions provided through RSON by rural health care providers working in the sites were nearly unanimously positive and reported to have led to increased stability and sustainability, including a larger volume of local deliveries, as participants in this study noted. Although targeted at lower volume sites, we anticipate that the benefits of dedicated funding experienced by rural sites in this study would be equally applicable to higher volume settings in any jurisdiction.

Data from this study, as well as the broader evaluation of the RSON initiative demonstrates improved health outcomes and provider sustainability due to the interventions. This would indicate the value of prioritizing these funding lines moving forward, in order to continue sustaining rural maternity services, guided by valuing a flexible funding model. This allows communities to self-prioritize needs and respond to local conditions in a way that reflects actual needs as opposed to an industrial approach where communities strive to fit the funding parameters.

The mechanisms of ‘scale and spread’ prioritized by RSON were to establish program value through the initial phase of implementation, framed by a robust evaluation plan. Additionally, program legacy was a high priority from the start and system structures (for example, data capture formats for local continuous quality improvement projects) were developed to endure past the initial phase. Most importantly, however, was the attention to relationship development with the Regional Health Authority planners to both ensure the program aligned with existing initiatives and priorities, but also to create pathways for further health authority level support at the end of the initial funding. Applying additional resources that prioritize activities that were previously ‘off the side of the desk’, was a proxy for valuing the work that was often done behind the scenes. This in turn became a lightning rod for cultural change which, we believe, will underscore enduring program elements into the future.

## Conclusion

Rural maternity services offering local procedural care in British Columbia benefitted from one-time, system level funding to support local provider teams, an assessment based on reflections of care providers and administrators in the communities and project data on health human resource staffing that reflects stability during the study time frame. In communities with a delivery threshold that would support local access to caesarean section, such funding appears to accrue disproportionate benefits and should be considered for inclusion in core rural health services funding schemes.

### Electronic supplementary material

Below is the link to the electronic supplementary material.

Supplementary Material 1

## Data Availability

The datasets generated and analyzed in this study are not publicly available to prevent subject identification, due to the small number of participants in a localized area. The data may be available from the corresponding author on reasonable request.

## List of abbreviations

RSON: Rural Surgical Obstetrical Network
OR: Operating Room
BC: British Columbia
CQI: Continuous Quality Improvement
FPESS: Family Physicians with Enhanced Surgical Skills
FPOSS: Family Physicians with Obstetrical Surgical Skills
FPA: Family Practice Anesthesia providers
RTVS: Real Time Virtual Supports
- GPESS: General Practitioner with Enhanced Surgical Skills
- OBs: - Obstetricians
RPT: Remote Presence Technology
FTE: Full-time equivalents
CHARLiE: Child Health Advice in ReaL-time Electronically
MaBAL: Maternity and Babies Advice Line
