# Supplementary material for: System interventions to support rural access to maternity care: an analysis of the rural surgical obstetrical networks program
Source: BMC Pregnancy Childbirth. 2023 Aug 29;23:621. doi: 10.1186/s12884-023-05898-7 (PMC10466771; doi:10.1186/s12884-023-05898-7)
Supplement: Supplementary file 1 — Supplementary Material 1 [file 12884_2023_5898_MOESM1_ESM.pdf]

RSON Global Interview Guide: Health Care Providers

Introductory questions:

- Can you describe your role in the community?

General questions:

- Can you describe the current status of maternity and/or surgical services in your community?
    - Please describe maternity services (e.g., number of local births; c-section coverage)
    - Please describe surgical services
- In your opinion, has your community benefited from RSON?
    - What are the major benefits of RSON?
    - Where has RSON fall short on?

COVID:

- How has COVID-19 continued to impact surgical and obstetrical services at your site this year?
- Please describe the effect of the ongoing COVID-19 pandemic on surgical services at your site specifically:
    - On patients
    - On health care providers
        - Has covid impacted your clinical coaching activities?
        - Has the OR closure led to increased work-life balance/anxiety etc.?
        - On the overall care supported in your community.
- Do you have any other comments about the impact of RSON on the sustainability of health services at your site?

Education/coaching

- What are some of the major clinical coaching activities that you have taken part in?
- What are some of the education and coaching opportunities that have been particularly useful?
    - Are there any coaching activities with regional centres who came into the community?
    - Do you have a chance to leave the community for coaching activities?
- Do you feel coaching activities improved relationships between peers and regional centres?
- Is there any activities that you wished to use RSON coaching funding but you were unable to use?

Visiting specialists:

- Are there any visiting specialists providing care in your community?
- How do visiting specialists contribute to the expansion of surgical or maternity services at your site?

RPT:

- Have you used RPT or RTVS technology?
  - What is your experience of the consults?
  - Has it been useful?
  - Has RPT been helpful during simulations? Was it easy to incorporate?
- What are some of the benefits of RPT (compared to calling personal contacts for consult?)
- IS RPT being used to conduct pre-operative screening in your community?

Nursing/ CQI:

- Can you describe the sustainability of nursing staff?
  - Do you see the need for a dedicated nursing line for maternity care?
- What kind of additional support from RSON would you like to see to support nurses?
- Do you think CQI projects are valuable?
- What is your experience with CQI?
  - Does it make sense for CQI work to be done by nurses?
- What other CQI projects do you hope to do before RSON ends?

Team culture:

- Have you used RSON funding for team-based or social activities such as lunches?
- How would you describe the current team culture?
  - Has RSON led to the shift in team culture?
- Can you describe your relationship with the regional referral outreach surgeons?
  - Has your relationship change over time?

Maternity services:

- Can you describe the maternity model in your community?
- Has RSON led to improvements in maternity care at your site?
  - Do you think gynecological needs are met?
- Do you feel like you're getting a high enough volume of deliveries to maintain your skillsets?
- Could you describe the relationship between surgical services and maternity care in at your site?

Post-RSON and succession planning:

- Do you have any other comments about the impact of RSON on the sustainability of health services at your site?
- Has your community progressed in succession planning for post-RSON? In what way? What is the collective capacity at your site right now to do this?
    - Do you feel the Health Authority is supportive?
- What are some of the goals or programs that you wish to keep or continue?
- What are some of the challenges you anticipate transitioning out of RSON?
    - Do you anticipate any dangers or threats to the sustainability of surgical and maternity services in your community?
    -
